# Health Risk in a Geographic Area of Thailand with Endemic Cadmium Contamination: Focus on Albuminuria

**DOI:** 10.3390/toxics11010068

**Published:** 2023-01-11

**Authors:** Soisungwan Satarug, David A. Vesey, Glenda C. Gobe, Supabhorn Yimthiang, Aleksandra Buha Đorđević

**Affiliations:** 1 Kidney Disease Research Collaborative, Translational Research Institute, Brisbane 4102, Australia; 2 Department of Nephrology, Princess Alexandra Hospital, Brisbane 4102, Australia; 3 School of Biomedical Sciences, The University of Queensland, Brisbane 4072, Australia; 4 NHMRC Centre of Research Excellence for CKD QLD, UQ Health Sciences, Royal Brisbane and Women’s Hospital, Brisbane 4029, Australia; 5 Occupational Health and Safety, School of Public Health, Walailak University, Nakhon Si Thammarat 80160, Thailand; 6 Department of Toxicology “Akademik Danilo Soldatović”, University of Belgrade-Faculty of Pharmacy, 11000 Belgrade, Serbia

**Keywords:** albuminuria, albumin-to-creatinine ratio, BMDL, BMDU, cadmium, creatinine clearance, creatinine excretion, estimated glomerular filtration rate, eGFR, toxicity threshold level

## Abstract

An increased level of cadmium (Cd) in food crops, especially rice is concerning because rice is a staple food for over half of the world’s population. In some regions, rice contributes to more than 50% of the total Cd intake. Low environmental exposure to Cd has been linked to an increase in albumin excretion to 30 mg/g creatinine, termed albuminuria, and a progressive reduction in the estimated glomerular filtration rate (eGFR) to below 60 mL/min/1.73 m^2^, termed reduced eGFR. However, research into albuminuria in high exposure conditions is limited. Here, we applied benchmark dose (BMD) analysis to the relevant data recorded for the residents of a Cd contamination area and a low-exposure control area. We normalized the excretion rates of Cd (E_Cd_) and albumin (E_alb_) to creatinine clearance (C_cr_) as E_Cd_/C_cr_ and E_alb_/C_cr_ to correct for differences among subjects in the number of surviving nephrons. For the first time, we defined the excretion levels of Cd associated with clinically relevant adverse kidney health outcomes. E_alb_/C_cr_ varied directly with E_Cd_/C_cr_ (β = 0.239, *p* < 0.001), and age (β = 0.203, *p* < 0.001), while normotension was associated with lower E_alb_/C_cr_ (β = −0.106, *p* = 0.009). E_Cd_/C_cr_ values between 16.5 and 35.5 ng/L of the filtrate were associated with a 10% prevalence of albuminuria, while the E_Cd_/C_cr_ value of 59 ng/L of the filtrate was associated with a 10% prevalence of reduced eGFR. Thus, increased albumin excretion and eGFR reduction appeared to occur at low body burdens, and they should form toxicity endpoints suitable for the calculation of health risk due to the Cd contamination of food chains.

## 1. Introduction

Cadmium (Cd) is detectable in virtually all food types and is becoming a toxic metal pollutant of public health concern worldwide [1,2,3]. Foods that are frequently consumed in large quantities such as rice, potatoes, wheat, leafy salad vegetables, and other cereal crops are the most significant dietary sources of Cd [4,5,6,7]. A rice Cd content of 0.27 mg/kg was associated with kidney and bone damage similar to those seen in itai-itai disease patients [8]. A lifetime Cd intake ≥ 1 g, which is half of the officially tolerable intake level, was associated with a 49% increase in mortality from kidney failure among women after adjustment for potential confounders [9]. These findings indicate that the Codex maximally permissible Cd level in rice of 0.4 mg/kg, and the lifetime Cd intake of 2 g, as suggested by the Joint Food and Agriculture Organization and World Health Organization (FAO/WHO) Expert Committee on Food Additives and Contaminants (JECFA), are not protective of human health [10,11]. New health guidance values need to be established for this toxic metal as well as public measures to minimize the Cd contamination of food chains.

Albuminuria is diagnosed when the excretion of albumin, measured as the albumin-to-creatinine ratio, rises to levels above 20 and 30 mg/g creatinine in men and women, respectively [12,13,14]. The persistence of albuminuria for at least three months is a diagnostic criterion of chronic kidney disease (CKD). A progressive decrease in the estimated glomerular filtration rate (eGFR) below 60 mL/min/1.73 m^2^, termed reduced eGFR, is also a diagnostic criterion of CKD [12,13,14].

Low environmental exposure to Cd experienced by participants of the U.S. National Health and Nutrition Examination Survey (NHANES) undertaken between 1999 and 2016 has been linked to albuminuria and reduced eGFR [15,16,17,18]. A urinary Cd concentration of 0.22 μg/L was associated with the increased excretion of albumin [19]. A urinary Cd excretion rate of 0.27 µg/g of creatinine was associated with a 58% increase in the risk of albuminuria in a Spanish population [20]. Cd excretion rates >1.72 µg/g of creatinine were associated with an elevated excretion of albumin in the residents of Shanghai [21]. Given that albuminuria and reduced eGFR were observed at a low Cd body burden, these signs of Cd toxicity are suitable for the derivation of an acceptable exposure level. 

A population exposed to a wide range of Cd doses is required to establish a clear dose–response relationship from which an acceptable dietary exposure level is reliably estimated. The Mae Sot District in western Thailand appeared to be ideal because it was an area where environmental Cd pollution was endemic [22,23,24]. This geographic area provided a well-circumscribed population of people with the same level of exposure that would enable one to discern the heath impact of excessive Cd ingestion [25,26,27]. More than 40% of residents aged ≥40 years were found to be at risk of Cd-induced toxic injury to tubular epithelial cells and Cd-induced defective tubular re-absorptive function [25]. Furthermore, the level of exposure among the Mae Sot residents appeared to be moderate enough to be likely experienced by many populations, who will carry the same risks, but they are “scattered” in many different places. Thus, a very large sample size would be needed to recruit enough number of those at risk of Cd-toxicity to a study. 

In high-exposure situations, tubular proteinuria, evident from an increased excretion of a low-molecular-weight protein, β_2_-microglobulin (β_2_M), is most frequently investigated [25,26,27]. In comparison, albuminuria has rarely been studied. Thus, in the present study, we aimed to characterize albumin excretion (E_alb_) and eGFR in relation to Cd exposure, measured as Cd excretion (E_Cd_), age, sex, smoking, and the presence of diabetes and hypertension. We also aimed to identify the benchmark dose (BMD) of Cd-induced albuminuria and Cd-induced eGFR reduction. The BMD is defined as a dose level, derived from an estimated dose–response curve, associated with a specified change in response, termed a benchmark response (BMR) [28,29,30]. BMD corrects some of the shortcomings of the no-adverse-effect level (NOAEL), and it has increasingly been used as the point of departure (POD) to derive health guidance values [28,29]. The lower 95% confidence bound of the BMD, termed a BMDL value derived from a continuous endpoint at 5% BMR, has been viewed as an NOAEL equivalent [28,29,30].

## 2. Materials and Methods

### 2.1. Study Subjects

Data were from 603 participants (400 females and 203 males) enrolled in studies conducted in a high-exposure area of the Mae Sot District, Tak Province [31] and a low-exposure location in Nakhon-Si-Thammarat Province [32]. As a prospective cohort study in Japan [9] observed an increased mortality from kidney disease, especially in women, more females were recruited to the present study than males. The study protocol for the Mae Sot group was approved by the Institutional Ethical Committees of Chiang Mai University and the Mae Sot Hospital. The study protocol for the Nakhon Si Thammarat group was approved by the Office of the Human Research Ethics Committee of Walailak University.

All participants gave informed consent prior to participation. They had lived at their current addresses for at least 30 years. Exclusion criteria were pregnancy, breast-feeding, a history of metal work, and a hospital record or physician’s diagnosis of an advanced chronic disease. All participants had presumably acquired Cd from the environment, given that occupational exposure was an exclusion criterion. Diabetes was defined as fasting plasma glucose levels ≥ 126 mg/dL (https://www.cdc.gov/diabetes/basics/getting-tested.html (accessed on 9 January 2023)) or a physician’s prescription of anti-diabetic medications. Hypertension was defined as systolic blood pressure ≥140 mmHg, diastolic blood pressure ≥ 90 mmHg [33], a physician’s diagnosis, or prescription of anti-hypertensive medications.

### 2.2. Collection and Analysis of Blood and Urine Samples

To enable normalizing excretion rates of Cd and albumin (E_Cd_ and E_alb_) to C_cr_, sampling of simultaneous blood and urine is required. Thus, second morning urine samples were collected after an overnight fast, and whole blood samples were obtained within 3 h after the urine sampling. Aliquots of urine, whole blood, and plasma were stored at −20 or −80 °C for later analysis. The assay for urine and plasma concentrations of creatinine ([cr]_u_ and [cr]_p_) was based on the Jaffe reaction. The assay of urinary albumin ([alb]_u_) was based on an immunoturbidimetric method. 

For the Mae Sot group, urinary Cd concentration ([Cd]_u_) was determined using atomic absorption spectrophotometer (AAS) (Shimadzu Model AA-6300, Kyoto, Japan). Urine standard reference material No. 2670 (National Institute of Standards, Washington, DC, USA) was used for quality assurance and control purposes. The LOD of Cd quantitation, defined as 3 times the standard deviation of blank measurements, was 0.06 µg/L. None of the urine samples contained [Cd]_u_ below the detection limit. 

For the Nakhon-Si-Thammarat group, [Cd]_u_ was determined using the GBC System 5000 Graphite Furnace AAS (GBC Scientific Equipment, Hampshire, IL, USA). An instrumental metal analysis was calibrated with the multi-element standards (Merck KGaA, Darmstadt, Germany). Reference urine metal control levels 1, 2, and 3 (Lyphocheck, Bio-Rad, Hercules, CA, USA) were used for quality control, analytical accuracy, and precision assurance. The analytical accuracy of metal detection was checked using an external quality assessment every 3 years. The LOD of urine Cd was 0.1 µg/L. When [Cd]_u_ was below its detection limit, the Cd concentration assigned was the detection limit divided by the square root of 2 [34].

### 2.3. Estimated Glomerular Filtration Rates (eGFRs)

The GFR is the product of nephron number and mean single nephron GFR, and in theory, the GFR is indicative of nephron function [12,13,14]. In practice, the GFR is estimated from established chronic kidney disease–epidemiology collaboration (CKD-EPI) equations and is reported as eGFR [14]. 

Male eGFR = 141 × [plasma creatinine/0.9]^Y^ × 0.993^age^, where Y = −0.411 if [cr]_p_ ≤ 0.9 mg/dL, and Y = −1.209 if [cr]_p_ > 0.9 mg/dL. Female eGFR = 144 × [plasma creatinine/0.7]^Y^ × 0.993^age^, where Y = −0.329 if [cr]_p_ ≤ 0.7 mg/dL, and Y = −1.209 if [cr]_p_ > 0.7 mg/dL. For dichotomous comparisons, CKD was defined as eGFR ≤ 60 mL/min/1.73 m^2^. CKD stages 1, 2, 3a, 3b, 4, and 5 corresponded to eGFR of 90–119, 60–89, 45–59, 30−44, 15–29, and <15 mL/min/1.73 m^2^, respectively. 

### 2.4. Normalization of E_Cd_ to E_cr_ and C_cr_

E_x_ was normalized to E_cr_ as [x]_u_/[cr]_u_, where x = Cd; [x]_u_ = urine concentration of x (mass/volume); and [cr]_u_ = urine creatinine concentration (mg/dL). The ratio [x]_u_/[cr]_u_ was expressed in μg/g of creatinine. 

E_x_ was normalized to C_cr_ as E_x_/C_cr_ = [x]_u_[cr]_p_/[cr]_u_, where x = Cd; [x]_u_ = urine concentration of x (mass/volume); [cr]_p_ = plasma creatinine concentration (mg/dL); and [cr]_u_ = urine creatinine concentration (mg/dL). E_x_/C_cr_ was expressed as the excretion of x per volume of filtrate. It corrects for differences in number of surviving nephrons among study subjects, and it depicts an amount of Cd and albumin excreted per volume of filtrate, which is at least roughly related to amount of Cd and albumin excreted per nephron [35]. 

### 2.5. Benchmark Dose Computation and Benchmark Response (BMR) Setting

The web-based PROAST software version 70.1 (https://proastweb.rivm.nl) (accessed on 22–29 July 2022) was employed to compute the BMD figures for Cd exposure as E_Cd_/E_cr_ or E_Cd_/C_cr_ associated with E_alb_ and eGFR. The BMDL value obtained from setting the BMR at 5% for continuous endpoints could be viewed as POD [28,29,30]. Data were fitted to multiple dose–response models that included inverse exponential, natural logarithmic, exponential, and Hill models. Model averaging was used to account for uncertainty.

The BMR was set at 5 and 10% increases in the prevalence of the following quantal endpoints: eGFR ≤ 60 mL/min/1.73 m^2^, E_alb_/E_cr_ ≥ 20 mg/g creatinine for men and ≥30 mg/g creatinine for women, and E_alb_/C_cr_ × 100 ≥ 20 mg/dL filtrate for men and ≥30 mg/dL filtrate for women. Data were fitted to several models that included two-stage, logarithmic logistic, Weibull, logarithmic probability, gamma, exponential, and Hill models.

The BMDL and BMDU corresponded to the lower bound and upper bound of the 95% confidence interval (CI) of BMD. The BMDL/BMDU were from model averaging using bootstrap with 200 repeats [36]. A wider BMDL-BMDU difference indicates a higher statistical uncertainty in the dataset [28,37,38,39].

### 2.6. Statistical Analysis

Data were analyzed using IBM SPSS Statistics 21 (IBM Inc., New York, NY, USA). To identify departures of continuous variables from a normal distribution, the one-sample Kolmogorov–Smirnov test was used. A logarithmic transformation was applied to variables that showed rightward skewing before they were analyzed parametric statistics. Differences in means among three residential groups were assessed using the Kruskal–Wallis test. The Mann–Whitney U test was used to compare mean differences between two eGFR groups. Differences in percentage and prevalence data were determined using the chi-square test. Univariate/covariance analysis with Bonferroni correction in multiple comparisons was employed to obtain the mean albumin excretion values adjusted for covariates and interaction in groups of subjects The logistic regression analysis was used to determine the prevalence odds ratio (POR) for albuminuria and reduced eGFR in relation to six independent variables: age, BMI, gender, smoking, hypertension, and Cd exposure measures (E_Cd_). We employed two models in each logistic regression analysis: model 1 incorporated log(E_Cd_/E_cr_) or E_Cd_/E_cr_ quartile; and model 2 incorporated log(E_Cd_/C_cr_) or E_Cd_/C_cr_ quartile. All other independent variables in models 1 and 2 were identical. For all tests, *p*-values ≤ 0.05 for two-tailed tests were assumed to indicate statistical significance.

## 3. Results

### 3.1. Study Subjects Stratified by Residential Location

The descriptive characteristics of the study subjects are provided in Table 1.

Among 603 subjects, 75 (12.4%) were residents of a low-exposure area (Pakpoon municipality), and 313 (51.9%) and 215 (35.6%) were residents of two areas of the Mae Sot District. The mean E_Cd_/E_cr_ in the Pakpoon group was 5.4- and 10.3-fold lower than those of the Mae Sot 1 and 2, respectively. 

The overall mean age was 52.4 years, and 44.9% were smokers, including those who had stopped less than 10 years ago. The percentages of females, hypertension, and diabetes were 66.3, 37.1, and 1.8%, respectively. The Pakpoon group was the oldest (mean age 61), which was 4 and 14 years older than the Mae Sot 1 and Mae Sot 2 groups, respectively. The overall percentage of reduced eGFR was 11.4%. The % of reduced eGFR in the Mae Sot 2 group of 23.7% was the highest, compared to 9.3 and 3.5% in the Pakpoon and Mae Sot 1 groups, respectively (*p* < 0.001).

The percentages of albuminuria according to E_cr_- and C_cr_-normalized data were 13.6 and 12.8%, respectively. The mean eGFR, BMI, urinary and plasma creatinine, urinary Cd and urinary albumin excretion were all statistically different across the three residential groups (*p* < 0.05). 

### 3.2. Study Subjects Stratified by Sex and eGFR 

The percentages of reduced eGFR among 203 males and 400 females were 13.8 and 10.2%, respectively (Table 2).

For the male group, the percentage of smoking did not differ (77.7% vs. 85.7%). The % of diabetes among males with reduced eGFR was 9% higher (*p* = 0.009). Half of the males in the reduced eGFR group had albuminuria (*p* < 0.001). The mean BMI did not differ nor did the percentage of hypertension. With the exception of urine creatinine, the mean values of all of the other continuous variables were higher in the reduced eGFR group.

For the female group, the percentage of smoking was higher in those with reduced eGFR (41.5% vs. 26.2%, *p* = 0.038). The percentages of diabetes and hypertension did not differ. In contrast, 29.3% of females in the reduced eGFR group had albuminuria, compared with 9.7% of those who had an eGFR above 60 mL/min/1.73 m^2^ (*p* < 0.001). With the exception of BMI and urine creatinine, the mean values for all of the other continuous variables were higher in the reduced eGFR group.

### 3.3. Multiple Regression Analysis of Albumin Excretion

The associations of albumin excretion and seven independent variables are shown for all subjects, including males and females (Table 3).

We employed two models: E_alb_ and E_Cd_ were incorporated as log[(E_alb_/E_cr_) × 10^3^] and log [(E_Cd_/E_cr_) × 10^3^] in model 1 and as log[(E_alb_/C_cr_) × 10^4^] and log [(E_Cd_/C_cr_) × 10^5^] in model 2. All other independent variables in models 1 and 2 were identical. 

In model 1, seven independent variables accounted for 6.7, 10.2, and 4.6% of the variation in E_alb_/C_cr_ in all subjects (*p* < 0.001), males (*p* < 0.001), and females (*p* < 0.001), respectively. In all subjects, higher E_alb_/E_cr_ were associated with older age (β = 0.162, *p* < 0.001) and higher E_Cd_/E_cr_ (β = 0.186, *p* < 0.001), while normotension was associated with lower E_alb_/E_cr_ (β = −0.106, *p* = 0.011). An association between E_alb_/E_cr_ and E_Cd_/E_cr_ was stronger in males (β = 0.279, *p* < 0.001) than females (β = 0.130, *p* = 0.020).

In model 2, seven independent variables accounted for 10.5, 16.4, and 7% of the variation in E_alb_/C_cr_ in all subjects (*p* < 0.001), males (*p* < 0.001), and females (*p* < 0.001), respectively. In all subjects, higher E_alb_/C_cr_ were associated with older age (β = 0.203, *p* < 0.001) and higher E_Cd_/C_cr_ (β = 0.239, *p* < 0.001), while normal blood pressure was weakly associated with lower E_alb_/C_cr_ (β = −0.106, *p* = 0.009). An association between E_alb_/C_cr_ and E_Cd_/C_cr_ was stronger in males (β = 0.342, *p* < 0.001) than females (β = 0.174, *p* = 0.001).

### 3.4. Dose–Effect Relationship between E_Cd_ and E_alb_

**Figure 1:**
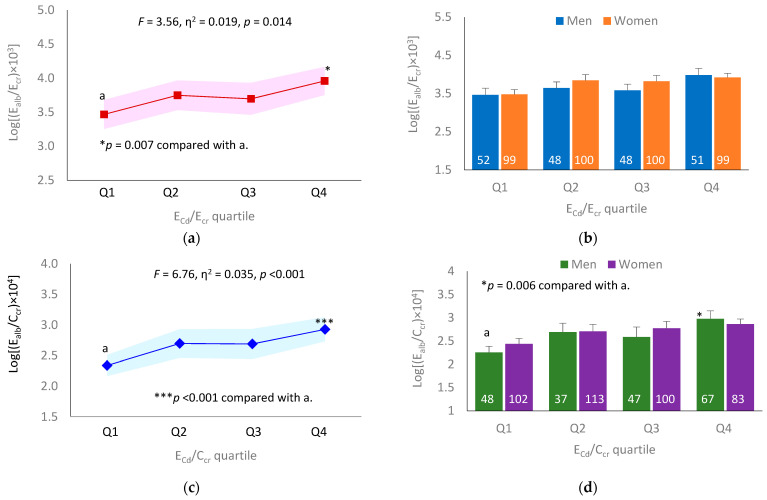
Comparing albumin excretion levels across cadmium excretion quartiles. (**a**) Mean E_alb_/E_cr_ values with variances across E_Cd_/E_cr_ quartiles 1−4; (**b**) mean E_alb_/E_cr_ values in men and women in E_Cd_/E_cr_ quartiles 1−4; (**c**) mean E_alb_/C_cr_ values with variances across E_Cd_/C_cr_ quartiles 1−4; (**d**) mean E_alb_/C_cr_ in men and women in E_Cd_/C_cr_ quartiles 1−4. Mean values were obtained using univariate analysis with adjustment for covariates.

Figure 1 graphically depicts the results of a covariance analysis of E_alb_ in subjects grouped by E_Cd_ quartiles.

For E_cr_-normalized data, E_Cd_ contributed to 1.9% of the variation in E_alb_ in all subjects (*F* = 3.56, *p* = 0.014) (Figure 1a). This effect of E_Cd/_E_cr_ was insignificant in separate males and females (Figure 1b). 

For C_cr_-normalized data, E_Cd_ contributed to 3.5% of the variation in E_alb_ in all subjects (*F* = 6.76, *p* < 0.001) (Figure 1c). This effect of E_Cd_/C_cr_ remained significant in males (Figure 1d).

### 3.5. Logistic Regression Analysis of Reduced eGFR

The results of an analysis of risk factors for reduced eGFR are shown in Table 4.

In model 1, the prevalence odds ratio (POR) for reduced eGFR was inversely associated with age (β −0.123, *p* < 0.001) and the E_Cd_/E_cr_ quartiles. Compared with E_Cd_/E_cr_ quartile 1, the POR for reduced eGFR increased by 4.4- and 4.2-fold in E_Cd_/E_cr_ quartile 3 (*p* = 0.002) and E_Cd_/E_cr_ quartile 4 (*p* = 0.001), respectively. An increase in the POR for reduced eGFR among those in E_Cd_/E_cr_ quartile 2 was insignificant (*p* = 0.085). 

In model 2, the POR for reduced eGFR was inversely associated with age (β = −0.131, *p* < 0.001) and the E_Cd_/C_cr_ quartiles. Compared with E_Cd_/C_cr_ quartile 1, the POR for reduced eGFR increased by 5.4-, 4.8-, and 23.4-fold in those in E_Cd_/C_cr_ quartile 2 (*p* < 0.002), E_Cd_/C_cr_ quartile 3 (*p* < 0.001), and E_Cd_/C_cr_ quartile 4 (*p* < 0.001), respectively. 

### 3.6. BMD Analysis of Albuminuria and Reduced eGFR according to E_cr_-Normalized Data

All dose–response model fittings are provided in the Supplemental Materials (Figures S1–S4). A summary of the results obtained from the BMD modeling is provided in Table 5.

As the data in Table 5 indicate, the BMDL/BMDU values of E_Cd_/E_cr_ producing a 5% increase of E_alb_/E_cr_ could not be estimated. Similarly, the quantal dose–effect analyses did not yield BMDL/BMDU values for Cd-induced albuminuria. In contrast, the BMDL/BMDU values of E_Cd_/E_cr_ were identified for the eGFR endpoint. In women, the respective BMDL values of E_Cd_/E_cr_ corresponding to a 5% reduction of eGFR and 5 and 10% increases in the prevalence of reduced eGFR were 6.82, 1.93, and 5.31 µg/g of creatinine. These female BMDL values of E_Cd_/E_cr_ were higher than those derived for men of 2.07, 1.47, and 3.92 µg/g of creatinine, respectively. 

### 3.7. BMD Analysis of Albuminuria and Reduced eGFR according to C_cr_-Normalized Data

All dose-response model fittings are provided in the Supplemental Material (Figures S5–S8). A summary of results obtained from the BMD modeling is provided in Table 6.

As the data in Table 6 indicate, the BMDL/BMDU values of E_Cd_/C_cr_ could not be reliably estimated when a 5% increase in E_alb_/C_cr_ was an endpoint. A quantal dose–effect analysis of a 5% increase in the prevalence of albuminuria yielded the E_Cd_/C_cr_ BMDL/BMDU values for men only. The BMDL/BMDU values of E_Cd_/C_cr_ corresponding to a 10% increase in albuminuria prevalence were obtained for both men and women. For the eGFR endpoint, the BMDL/BMDU values of E_Cd_/C_cr_ were identified with a high degree of statistical certainty. In women, the respective BMDL values of E_Cd_/C_cr_ × 100 corresponding to a 5% reduction of eGFR and 5 and 10% increases in the prevalence of reduced eGFR were 2.15, 3.33, and 5.88 µg/L of filtrate. These BMDL values of E_Cd_/C_cr_ were nearly identical to the BMDL values of E_Cd_/C_cr_ × 100 derived for men of 2.15, 3.22, and 5.61 µg/L of filtrate.

## 4. Discussion

In the present study, we derived simultaneously the BMDL values of Cd excretion levels from two CKD diagnostic criteria, albuminuria and reduced eGFR. We know of other studies that have identified the BMDL value of Cd excretion based on an albuminuria endpoint. In quantal dose–effect analyses, the prevalence of albuminuria was likely to be smaller than 10% at E_Cd_/C_cr_ values of 16.5 and 35.5 ng/L of filtrate in men and women, respectively (Figures S7 and S8). In comparison, the prevalence of reduced eGFR was likely to be smaller than 10% at E_Cd_/C_cr_ values of 56.1 and 58.8 ng/L of filtrate in men and women, respectively. 

The evidence that Cd may have increased albumin excretion among study subjects comes from a multiple regression analysis, where E_alb_/C_cr_ was independently associated E_Cd_/C_cr_ (β = 0.239, Table 3). Additional data from a regression analysis suggested a stronger effect of Cd on albumin excretion in men (β = 0.342) than women (β = 0.174). This gender differential association between E_alb_/C_cr_ and E_Cd_/C_cr_ was confirmed using a covariance analysis, where mean E_alb_/C_cr_ increased with the E_Cd_/C_cr_ quartiles, especially in men after adjustment for covariates that included age and BMI (Figure 1c,d).

The current evidence suggests that the bulk of protein/albumin (~80%) in the glomerular ultrafiltrate is retrieved in the S1 sub-segment of the proximal tubule, where the receptor-mediated endocytosis involving the megalin/cubillin system is expressed [40,41,42,43]. Reabsorption of albumin also occurs in the distal tubule and collecting duct, where the process is mediated by the NGAL/lipocalin-2 receptor system [43,44,45]. An experimental work showed that Cd disabled the cubillin/megalin receptor system of albumin endocytosis, leading to albuminuria [46]. In another study, Cd diminished the expression of megalin and ClC5 channels [47]. Cd may also increase glomerular permeability to albumin as shown in another study, where a non-cytotoxic concentration of Cd (1 µM) increased the permeability of human renal glomerular endothelial cells in monolayers and caused the redistribution of the adherens junction proteins, vascular endothelial cadherin and β-catenin [48,49]. Further research is required to dissect the glomerular and tubular causes of Cd-induced albuminuria.

It is noteworthy that the BMDL values of the Cd excretion levels producing an increment of E_alb_ and albuminuria prevalence were obtained only when E_alb_ and E_Cd_ were normalized to C_cr_ (Table 6, Figures S4–S8). High variance introduced to the dataset by E_cr_-normalization contributed to a failure to obtain reliable BMDL/BMDU values of E_Cd_ from an albuminuria endpoint (Table 5, Figures S1–S4). Although normalization by E_cr_ corrects urine dilution, this practice adds to dataset variation that is unrelated to Cd exposure or albumin excretion. An effect of E_cr_ normalization was demonstrable in a covariance analysis, where the relationship between E_Cd_/E_cr_ and E_alb_/E_cr_ was absent (Figure 1a,b). In contrast, a Cd-dose-dependent increase in E_alb_/C_cr_ was evident (Figure 1c,d).

The Cd contamination in Mae Sot District occurred mainly in three subdistricts, Phrathat Phadaeng, Mae Tao, and Mae Ku, where high urinary Cd levels and signs of adverse effects on kidneys were observed in both children and adults [25,26,27]. The three Cd hot spots were irrigated by Mae Tao and Mae Ku creeks which branched off the Moei River, and the paddy soils and the produce, including rice, became contaminated from the use of irrigated water that was polluted by Cd [22,23,24]. The Cd content of the paddy soil samples (*n* = 154) ranged from 3.4 to 284 mg/kg, exceeding the standard of 0.15 mg/kg [24]. Approximately 20% of the rice samples (*n* = 159) collected from households in the Mae Tao subdistrict contained Cd exceeding the Codex standard of 0.4 mg/kg [24].

We inferred from the above data that rice was the main Cd exposure source among the subjects in Mae Sot 1 and Mae Sot 2, who had, on average, 5 to 10 times higher E_Cd_/E_cr_ than the Pakpoon group (Table 1). In a Japanese study, rice and its products constituted 40–50% of dietary Cd exposure in two groups of women living in two areas affected by Cd contamination [7]. The differences between the groups with respect to dietary Cd exposure were attributed mostly to the Cd levels in rice consumed in the two areas. In a Japan total diet study undertaken from 2013 to 2018, the mean dietary Cd exposure was 0.35 μg/kg bw/day, ranging between 0.25 and 0.45 μg/kg bw/day [6]. The percentage contribution to Cd intake from rice and its products, green vegetables, cereals, and seeds plus potatoes were 38, 17, and 11%, respectively. 

In a previous risk analysis conducted on Mae Sot residents, the BMDL values of E_Cd_/E_cr_ for a β_2_M endpoint were 6.9 and 8.1 μg/g creatinine in men and women, respectively [27]. In contrast, in the present study, the respective BMDL values of E_Cd_/E_cr_ corresponding to a 5% reduction of eGFR and 5 and 10% increases in the prevalence of reduced eGFR were 6.82, 1.93, and 5.31 µg/g of creatinine in women. These female BMDL values of E_Cd_/E_cr_ were higher than those derived from men of 2.07, 1.47, and 3.92 µg/g of creatinine, respectively. The discrepancies in the BMDL values of E_Cd_/E_cr_ for adverse effects on kidneys derived by us and Nishijo et al. (2014) are due to the different dose–effect models used, different assumptions, and different endpoints. To advance toxicological risk assessment and its application to public health, the standardization of BMD methods and normalization of excretion data to C_cr_ are imperative.

In theory, an acceptable level of environmental exposure to Cd should be derived from the most sensitive toxicity endpoint, which is the one with the lowest BMDL value [30]. Furthermore, as the basic mechanisms of Cd cytotoxicity should be the same, the BMDL values of Cd excretion rates (body burdens) that produce any adverse kidney outcomes can be expected to be the same for men and women. However, Cd excretion rates of 6.82 and 2.07 µg/g of creatinine were found to be BMDLs that produce a 5% reduction of eGFR in males and females, respectively (Table 5). The higher BMDL value of the Cd excretion level for eGFR decline in females than males could be attributable to the lower creatinine excretion levels among females, compared to males. In contrast, the BMDL value of E_Cd_/C_cr_ × 100 producing a 5% reduction in eGFR was 2.15 µg/L of filtrate and identical in men and women (Table 6).

## 5. Conclusions

The conventional method for adjusting the excretion rates of Cd and albumin to the excretion of creatine (E_cr_) incorporates a conceptual flaw that can be eliminated if the rates are normalized to creatinine clearance (C_cr_). The NOAEL equivalents of Cd accumulation levels corresponding to a discernable increase in the prevalence of albuminuria in the population can only be derived with a high degree of a statistical certainty from C_cr_-normalized data. The narrow difference between BMDU and BMDL also indicate the reliability of the NOAEL derived. Normalization to C_cr_ should replace E_cr_ adjustment in studies attempting to relate urine composition to Cd-induced albuminuria and estimate the health risk for Cd exposure.

## Figures and Tables

**Table 1:** Study subjects according to residential location.

Parameters	All Subjects *n* 603	Residential Location			*p*
		Pakpoon, *n* 75	Mae Sot 1, *n* 313	Mae Sot 2, *n* 215	
Females (%)	66.3	78.7	72.2	53.5	<0.001
Smoking (%)	44.9	10.7	40.9	62.8	<0.001
Diabetes (%)	1.8	0	0	5.1	<0.001
Hypertension (%)	37.1	42.7	46.0	22.3	<0.001
Age, years	52.4 ± 9.8	61.1 ± 8.7	47.2 ± 4.7	57.0 ± 11.1	<0.001
BMI, kg/m^2^	23.4 ± 4.0	24.6 ± 4.5	24.5 ± 3.5	21.4 ± 3.6	<0.001
eGFR ^a^, mL/min/1.73 m^2^	85 ± 20	78 ± 13	96 ± 16	72 ± 19	<0.001
eGFR range	20−131	40−105	32−127	20−131	
Reduced eGFR ^b^ (%)	11.4	9.3	3.5	23.7	<0.001
Plasma creatinine, mg/dL	0.90 ± 0.29	0.87 ± 0.16	0.79 ± 0.20	1.07 ± 0.35	<0.001
Urine creatinine, mg/dL	119.5 ± 70.9	96.4 ± 53.2	125.8 ± 78.8	118.4 ± 62.2	0.016
Urine albumin, mg/L	19.0 ± 53.1	16.0 ± 49.4	15.2 ± 37.5	25.6 ± 70.6	<0.001
Urine Cd, µg/L	7.37 ± 9.34	0.70 ± 1.09	5.89 ± 6.13	11.85 ± 12.28	<0.001
Normalized to E_cr_ as E_x_/E_cr_ ^c^					
E_alb_/E_cr_, mg/g creatinine	17.6 ± 45.7	14.7 ± 33.8	14.5 ± 40.6	23.2 ± 55.1	<0.001
E_alb_/E_cr_ ≥ 20 or 30 mg/g (%) ^d^	13.6	9.3	11.5	18.1	0.047
E_Cd_/E_cr_, µg/g creatinine	6.65 ± 6.61	1.00 ± 1.90	5.40 ± 4.51	10.43 ± 8.02	<0.001
Normalized to C_cr_ as E_x_/C_cr_ ^e^					
(E_alb_/C_cr_) × 100, mg/L filtrate	18.8 ± 56.8	12.6 ± 31.6	13.2 ± 43.9	29.1 ± 76.0	<0.001
(E_alb_/C_cr_) × 100 ≥ 20 or 30 mg/L ^f^ (%)	12.8	10.7	8.6	19.5	0.001
(E_Cd_/C_cr_) × 100, µg/L filtrate	6.33 ± 7.59	0.88 ± 1.66	4.25 ± 3.88	11.27 ± 9.89	<0.001

*n*, number of subjects; BMI, body mass index; eGFR, estimated glomerular filtration rate; E_x_, excretion of x; cr, creatinine; C_cr_, creatinine clearance; alb, albumin; Cd, cadmium; ^a^ eGFR was calculated using Chronic Kidney Disease Epidemiology Collaboration (CKD−EPI) equations [14]; ^b^ reduced eGFR was defined as eGFR ≤ 60 mL/min/1.73 m^2^; ^c^ E_x_/E_cr_ = [x]_u_/[cr]_u_; ^d^ for E_cr_-normalized dataset, albuminuria was defined as albumin-to-creatinine ratio ≥ 20 mg/g for men and ≥30 mg/g for women; ^e^ E_x_/C_cr_ = [x]_u_[cr]_p_/[cr]_u_, where x = Cd, alb [35]. Data for all continuous variables are arithmetic mean ± standard deviation (SD) values. ^f^ For C_cr_-normalized dataset, albuminuria was defined as E_alb_/C_cr_ × 100 ≥ 20 mg/L filtrate for men and ≥30 mg/L filtrate for women. Data for BMI are from 597 subjects; data for all other variables are from 603 subjects. For each test, *p* ≤ 0.05 identifies statistical significance, determined using Pearson chi-square test for % differences and the Kruskal–Wallis test for mean differences across three residential areas.

**Table 2:** Characterization of study subjects according to sex and eGFR levels.

Parameters	Males (*n* 203)		Females (*n* 400)	
	eGFR > 60, *n* 175	eGFR ≤ 60, *n* 28	eGFR > 60, *n* 359	eGFR ≤ 60, *n* 41
Smoking (%)	77.7	85.7	26.2	41.5 ^#^
Hypertension (%)	28.0	42.9	41.2	36.6
Diabetes (%)	1.7	10.7 *	1.1	2.4
Age, years	51.3 ± 9.2	67.6 ± 10.6 ***	50.9 ± 8.3	60.2 ± 11.0 ^###^
BMI, kg/m^2^	22.3 ± 3.2	20.9 ± 3.3	24.1 ± 4.1	23.5 ± 4.5
eGFR ^a^, mL/min/1.73 m^2^	89 ± 16	44 ± 12 ***	90 ± 16	50 ± 10 ^###^
Plasma creatinine, mg/dL	0.97 ± 0.16	1.70 ± 0.45 ***	0.76 ± 0.14	1.24 ± 0.29 ^###^
Urine creatinine, mg/dL	136.9 ± 63.5	144.7 ± 68.3	110.6 ± 73.7	105.3 ± 60.7
Urine albumin, mg/L	18.0 ± 45.4	61.5 ± 80.4 ***	15.2 ± 51.9	27.8 ± 59.9 ^##^
Urine Cd, µg/L	7.94 ± 10.65	16.42 ± 16.22 **	9.11 ± 11.90	10.49 ± 12.46 ^#^
Normalized to E_cr_ as E_x_/E_cr_ ^b^				
E_Alb_/E_cr_, mg/g creatinine	16.7 ± 49.1	53.7 ± 83.0 ***	14.1 ± 38.7	28.2 ± 42.5 ^##^
Albuminuria ^c^	12.0	50.0 ***	9.7	29.3 ^###^
E_Cd_/E_cr_, µg/g creatinine	6.20 ± 6.95	10.36 ± 7.07 ***	6.15 ± 5.67	10.36 ± 9.97 ^#^
Normalized to C_cr_ as E_x_/C_cr_ ^d^				
E_Alb_/C_cr_ × 100, mg/L filtrate	16.6 ± 48.8	105.1 ± 167.5 ***	11.3 ± 34.6	34.5 ± 51.3 ^###^
E_Cd_/C_cr_ × 100, µg/L filtrate	6.16 ± 6.98	17.98 ± 13.38 ***	4.74 ± 4.66	13.05 ± 13.25 ^###^

eGFR, estimated glomerular filtration rate; *n*, number of subjects; BMI, body mass index; E_x_, excretion of x; cr, creatinine; C_cr_, creatinine clearance; Cd, cadmium; ^a^ eGFR was determined using Chronic Kidney Disease Epidemiology Collaboration equations [14]. ^b^ E_x_/E_cr_ = [x]_u_/[cr]_u_; ^c^ E_x_/C_cr_ = [x]_u_[cr]_p_/[cr]_u_, where x = Cd or albumin [35]; ^d^ E_x_/C_cr_ = [x]_u_[cr]_p_/[cr]_u_, where x = Cd. Data for all continuous variables are arithmetic mean ± standard deviation (SD) values. Data for BMI values are from 597 subjects; data for all other variables are from 603 subjects. For each test, *p* ≤ 0.05 identifies statistical significance, determined using Pearson’s chi-square test for percentage differences and the Mann–Whitney U-test for mean differences between two eGFR groups. For the male group, * *p* = 0.009, ** *p* = 0.001, and *** *p* ≤ 0.001. For the female group, ^#^ *p* = 0.018−0.039, ^##^ *p* = 0.001−0.002, and ^###^ *p* ≤ 0.001.

**Table 3:** Multiple regression model analysis of albumin excretion.

Independent Variables/ Factors	Urinary Excretion of Albumin ^a^					
	All Subjects, *n* 603		Males, *n* 203		Females, *n* 400	
	β ^b^	*p*	β	*p*	β	*p*
*Model 1*						
Age, years	0.162	<0.001	0.173	0.022	0.137	0.011
BMI, kg/m^2^	0.036	0.426	0.014	0.852	0.041	0.450
Log [(E_Cd_/E_cr_) × 10^3^], µg/g creatinine	0.186	<0.001	0.279	<0.001	0.130	0.020
Diabetes	−0.057	0.158	−0.017	0.810	−0.086	0.083
Sex	−0.067	0.147	−	−	−	−
Hypertension	−0.106	0.011	−0.134	0.060	−0.087	0.086
Smoking	−0.089	0.063	−0.001	0.990	−0.122	0.020
Adjusted R^2^	0.067	<0.001	0.102	<0.001	0.046	<0.001
*Model 2*						
Age, years	0.203	<0.001	0.198	0.007	0.182	0.001
BMI, kg/m^2^	0.048	0.278	0.019	0.798	0.056	0.300
Log [(E_Cd_/C_cr_) × 10^5^], µg/L filtrate	0.239	<0.001	0.342	<0.001	0.174	0.001
Diabetes	−0.007	0.880	−0.017	0.809	−0.092	0.061
Sex	−0.060	0.132	−	−	−	−
Hypertension	−0.106	0.009	−0.137	0.047	−0.085	0.089
Smoking	−0.073	0.121	0.017	0.806	−0.113	0.029
Adjusted R^2^	0.105	<0.001	0.164	<0.001	0.070	<0.001

^a^ Albumin excretion was as log[(E_alb_/E_cr_) × 10^3^] in model 1 and log[(E_alb_/C_cr_) × 10^4^] in model 2; ^b^ β, standardized regression coefficients. Coding: female = 1, male = 2, hypertensive = 1, normotensive = 2, smoker = 1, non-smoker = 2. Data were generated from regression model analyses relating E_alb_ to seven independent variables listed in the first column. E_Cd_ was as log [E_Cd_/E_cr_) × 10^3^] in model 1 or log [E_Cd_/C_cr_) × 10^5^] in model 2. All other independent variables in models 1 and 2 were identical. For each test, *p*-values < 0.05 indicate a statistical significance association. β coefficient indicates the strength of an association of E_alb_ and an individual independent variable. Adjusted R^2^ indicates the proportion of the variation of E_alb_ attributable to all seven independent variables.

**Table 4:** Prevalence of reduced eGFR in relation to E_Cd_ quartiles and other variables.

Independent Variables/ Factors	Number of Subjects	Reduced eGFR ^a^				
		β Coefficients	POR	95% CI		*p*
		(SE)		Lower	Upper	
*Model 1*						
Age, years	597	−0.123 (0.015)	0.885	0.859	0.912	<0.001
BMI, kg/m^2^	597	−0.071 (0.038)	0.932	0.865	1.003	0.061
Diabetes	11	−0.780 (0.829)	0.458	0.090	2.325	0.346
Hypertension	224	−0.276 (0.319)	0.759	0.406	1.418	0.386
Sex (females)	398	−0.032 (0.355)	0.968	0.483	1.943	0.928
Smoking	269	−0.193 (0.361)	0.825	0.407	1.672	0.593
Quartile of E_Cd_/E_cr_ in µg/g creatinine						
Q1: 0.03−2.41	151	Referent				
Q2: 2.42−4.64	148	0.651 (0.377)	1.917	0.915	4.015	0.085
Q3: 4.65−8.36	148	1.470 (0.482)	4.349	1.692	11.183	0.002
Q4: 8.37−57.6	150	1.446 (0.440)	4.245	1.792	10.055	0.001
*Model 2*						
Age, years	597	−0.131 (0.017)	0.878	0.849	0.906	<0.001
BMI, kg/m^2^	597	−0.093 (0.041)	0.912	0.842	0.987	0.023
Diabetes	11	−0.450 (0.826)	0.638	0.126	3.218	0.586
Hypertension	224	−0.408 (0.334)	0.665	0.346	1.279	0.221
Sex (females)	398	−0.295 (0.375)	0.745	0.357	1.555	0.433
Smoking	269	0.012 (0.368)	1.012	0.492	2.083	0.974
Quartile of (E_Cd_/C_cr_) × 100, µg/L filtrate						
Q1: 0.03−1.95	150	Referent				
Q2: 1.96−3.88	150	1.683 (0.432)	5.382	2.310	12.543	<0.001
Q3: 3.89−7.68	147	1.573 (0.426)	4.820	2.090	11.115	<0.001
Q4: 7.69−63.2	150	3.154 (0.603)	23.429	7.179	76.464	<0.001

POR, prevalence odds ratio; S.E., standard error of mean; CI, confidence interval. Coding: female = 1, male = 2, hypertensive = 1, normotensive = 2, smoker = 1, non-smoker = 2. ^a^ Reduced eGFR was defined as eGFR ≤ 60 mL/min/1.73 m^2^. Data were generated from logistic regression analyses relating POR for reduced eGFR to seven independent variables. E_Cd_/E_cr_ quartiles were incorporated into model 1; E_Cd_/C_cr_ × 100 quartiles were incorporated into model 2. Other independent variables in models 1 and 2 were identical. Each β coefficient indicates an effect size of an individual independent variable. For all tests, *p* values < 0.05 indicate statistical significance.

**Table 5:** BMDL and BMDU values of E_Cd_/E_cr_ computed from albumin excretion and eGFR.

Endpoints	Males			Females		
	BMDL	BMDU	U/L	BMDL	BMDU	U/L
5% increase of E_alb_/E_cr_	4.16 × 10^−6^	2.29	5.5 × 10^5^	3.89 × 10^−4^	3.41	8.8 × 10^3^
5% increase in prevalence of albuminuria ^a^	3.06 × 10^−3^	36.7	1.2 × 10^2^	1.22 × 10^−2^	3.05 × 10^5^	2.5 × 10^7^
10% increase in prevalence of albuminuria	0.55	337	612	2.52	1.74 × 10^6^	6.7 × 10^5^
5% Decrease of eGFR	2.07	6.93	3.35	6.82	21.7	3.81
5% increase in prevalence of reduced eGFR ^b^	1.47	10.6	7.21	1.93	15.6	8.08
10% increase in prevalence of reduced eGFR	3.92	15.7	4.00	5.31	23.6	4.44

BMDL and BMDU values of E_Cd_/E_cr_ were as µg/g creatinine. CI, confidence interval; U/L, BMDU/BMDL ratio. U/L ≥ 100 indicated unreliability due to a high degree of statistical uncertainty. ^a^ Albuminuria was defined as urinary albumin-to-creatinine ratios ≥ 20 mg/g for men and ≥30 mg/g for women. ^b^ Reduced eGFR was defined as eGFR ≤ 60 mL/min/1.73 m^2^.

**Table 6:** BMDL and BMDU values of E_Cd_/C_cr_ computed from albumin excretion and eGFR endpoints.

Endpoints	Males			Females		
	BMDL	BMDU	U/L	BMDL	BMDU	U/L
5% increase of E_alb_/C_cr_ × 100	0.00053	1.11	2094	0.00357	2.09	585
5% increase in prevalence of albuminuria ^a^	0.163	13	80	0.718	154	214
10% increase in prevalence of albuminuria	1.65	20	12	3.55	212	60
5% Decrease of eGFR	2.15	6.71	3.12	2.15	6.56	3.05
5% increase in prevalence of reduced eGFR ^b^	3.22	9.64	2.99	3.33	9.2	2.76
10% increase in prevalence of reduced eGFR	5.61	13.4	2.39	5.88	12.9	2.19

BMDL and BMDU values of E_Cd_/E_cr_ × 100 were as µg/L filtrate. CI, confidence interval; U/L, BMDU/BMDL ratio. U/L ≥ 100 indicated unreliability due to a high degree of statistical uncertainty. ^a^ Albuminuria was defined as E_Alb_/C_cr_ × 100 ≥ 20 mg/L filtrate for men and ≥30 mg/L filtrate for women. ^b^ Reduced eGFR was defined as eGFR ≤ 60 mL/min/1.73 m^2^.

## Data Availability

All data are contained within this article.

## Supplementary Materials

The following supporting information can be downloaded at: https://www.mdpi.com/article/10.3390/toxics11010068/s1, Figure S1: Dose–effect relationship of E_Cd_/E_cr_ and E_alb_/E_cr_; Figure S2: Dose–effect relationship of E_Cd_/E_cr_ and eGFR; Figure S3: Dose–effect relationship of E_Cd_/Ecr and albuminuria prevalence; Figure S4: Dose–effect relationship of E_Cd_/E_cr_ and prevalence of reduced eGFR; Figure S5: Dose–effect relationship of E_Cd_/C_cr_ and E_alb_/C_cr_; Figure S6: Dose–effect relationship of E_Cd_/C_cr_ and eGFR; Figure S7: Dose–effect relationship of E_Cd_/C_cr_ and albuminuria prevalence; Figure S8: Dose–effect relationship of E_Cd_/C_cr_ and prevalence of reduced eGFR.

## References

[1] Satarug S., Vesey D.A., Gobe G.C. (2017). Current health risk assessment practice for dietary cadmium: Data from different countries. Food Chem. Toxicol..

[2] Fechner C., Hackethal C., Höpfner T., Dietrich J., Bloch D., Lindtner O., Sarvan I. (2022). Results of the BfR MEAL Study: In Germany, mercury is mostly contained in fish and seafood while cadmium, lead, and nickel are present in a broad spectrum of foods. Food Chem. X.

[3] Boon P.E., Pustjens A.M., Te Biesebeek J.D., Brust G.M.H., Castenmiller J.J.M. (2022). Dietary intake and risk assessment of elements for 1- and 2-year-old children in the Netherlands. Food Chem Toxicol..

[4] Arnich N., Sirot V., Rivière G., Jean J., Noël L., Guérin T., Leblanc J.-C. (2012). Dietary exposure to trace elements and health risk assessment in the 2nd French total diet study. Food Chem. Toxicol..

[5] Sand S., Becker W. (2012). Assessment of dietary cadmium exposure in Sweden and population health concern including scenario analysis. Food Chem. Toxicol..

[6] Watanabe T., Kataoka Y., Hayashi K., Matsuda R., Uneyama C. (2022). Dietary exposure of the Japanese general population to elements: Total diet study 2013–2018. Food Saf..

[7] Horiguchi H., Oguma E., Sasaki S., Miyamoto K., Hosoi Y., Ono A., Kayama F. (2020). Exposure assessment of cadmium in female farmers in cadmium-polluted areas in Northern Japan. Toxics.

[8] Nogawa K., Sakurai M., Ishizaki M., Kido T., Nakagawa H., Suwazono Y. (2017). Threshold limit values of the cadmium concentration in rice in the development of itai-itai disease using benchmark dose analysis. J. Appl. Toxicol..

[9] Nishijo M., Nogawa K., Suwazono Y., Kido T., Sakurai M., Nakagawa H. (2020). Lifetime cadmium exposure and mortality for renal diseases in residents of the cadmium-polluted Kakehashi River Basin in Japan. Toxics.

[10] Codex Alimentarius, CODEX STAN 193–1995, General Standard for Contaminants and Toxins in Food and Feed. http://www.fao.org/fileadmin/user_upload/livestockgov/documents/1_CXS_193e.pdf.

[11] JECFA (2011). In Proceedings of the Joint FAO/WHO Expert Committee on Food Additives and Contaminants, Seventy-Third Meeting, Geneva, Switzerland, 8–17 June 2010. Summary and Conclusions.

[12] Murton M., Goff-Leggett D., Bobrowska A., Garcia Sanchez J.J., James G., Wittbrodt E., Nolan S., Sörstadius E., Pecoits-Filho R., Tuttle K. (2021). Burden of chronic kidney disease by KDIGO categories of glomerular filtration rate and albuminuria: A Systematic review. Adv. Ther..

[13] Soveri I., Berg U.B., Björk J., Elinder C.G., Grubb A., Mejare I., Sterner G., Bäck S.E., SBU GFR Review Group (2014). Measuring GFR: A systematic review. Am. J. Kidney Dis..

[14] Levey A.S., Becker C., Inker L.A. (2015). Glomerular filtration rate and albuminuria for detection and staging of acute and chronic kidney disease in adults: A systematic review. JAMA.

[15] Ferraro P.M., Costanzi S., Naticchia A., Sturniolo A., Gambaro G. (2010). Low level exposure to cadmium increases the risk of chronic kidney disease: Analysis of the NHANES 1999–2006. BMC Publ. Health.

[16] Navas-Acien A., Tellez-Plaza M., Guallar E., Muntner P., Silbergeld E., Jaar B., Weaver V. (2009). Blood cadmium and lead and chronic kidney disease in US adults: A joint analysis. Am. J. Epidemiol..

[17] Li Y.S., Ho W.C., Caffrey J.L., Sonawane B. (2014). Low serum zinc is associated with elevated risk of cadmium nephrotoxicity. Environ. Res..

[18] Madrigal J.M., Ricardo A.C., Persky V., Turyk M. (2018). Associations between blood cadmium concentration and kidney function in the U.S. population: Impact of sex, diabetes and hypertension. Environ. Res..

[19] Zhu X.J., Wang J.J., Mao J.H., Shu Q., Du L.Z. (2019). Relationships of cadmium, lead, and mercury levels with albuminuria in US adults: Results from the National Health and Nutrition Examination Survey Database, 2009–2012. Am. J. Epidemiol..

[20] Grau-Perez M., Pichler G., Galan-Chilet I., Briongos-Figuero L.S., Rentero-Garrido P., Lopez-Izquierdo R., Navas-Acien A., Weaver V., García-Barrera T., Gomez-Ariza J.L. (2017). Urine cadmium levels and albuminuria in a general population from Spain: A gene-environment interaction analysis. Environ. Int..

[21] Feng X., Zhou R., Jiang Q., Wang Y., Chen C. (2022). Analysis of cadmium accumulation in community adults and its correlation with low-grade albuminuria. Sci. Total Environ..

[22] Simmons R.W., Noble A.D., Pongsakul P., Sukreeyapongse O., Chinabut N. (2009). Cadmium-hazard mapping using a general linear regression model (Irr-Cad) for rapid risk assessment. Environ. Geochem. Health.

[23] Simmons R.W., Pongsakul P., Saiyasitpanich D., Klinpholap S. (2005). Elevated levels of cadmium and zinc in paddy soils and elevated levels of cadmium in rice grain downstream of a zinc mineralized area in Thailand: Implications for public health. Environ. Geochem. Health.

[24] Suwatvitayakorn P., Ko M.S., Kim K.W., Chanpiwat P. (2020). Human health risk assessment of cadmium exposure through rice consumption in cadmium-contaminated areas of the Mae Tao sub-district, Tak, Thailand. Environ. Geochem. Health.

[25] Nishijo M., Suwazono Y., Ruangyuttikarn W., Nambunmee K., Swaddiwudhipong W., Nogawa K., Nakagawa H. (2014). Risk assessment for Thai population: Benchmark dose of urinary and blood cadmium levels for renal effects by hybrid approach of inhabitants living in polluted and non-polluted areas in Thailand. BMC Publ. Health.

[26] Swaddiwudhipong W., Mahasakpan P., Jeekeeree W., Funkhiew T., Sanjum R., Apiwatpaiboon T., Phopueng I. (2015). Renal and blood pressure effects from environmental cadmium exposure in Thai children. Environ Res..

[27] Swaddiwudhipong W., Nguntra P., Kaewnate Y., Mahasakpan P., Limpatanachote P., Aunjai T., Jeekeeree W., Punta B., Funkhiew T., Phopueng I. (2015). Human health effects from cadmium exposure: Comparison between persons living in cadmium-contaminated and non-contaminated areas in northwestern Thailand. Southeast Asian J. Trop. Med. Publ. Health.

[28] EFSA Scientific Committee (2017). Update: Use of the benchmark dose approach in risk assessment. EFSA J..

[29] Wong C., Roberts S.M., Saab I.N. (2022). Review of regulatory reference values and background levels for heavy metals in the human diet. Regul. Toxicol. Pharmacol..

[30] Moffett D.B., Mumtaz M.M., Sullivan D.W., Whittaker M.H., Nordberg G., Costa M. (2022). Chapter 13, General Considerations of Dose-Effect and Dose-Response Relationships. Handbook on the Toxicology of Metals.

[31] Satarug S., Swaddiwudhipong W., Ruangyuttikarn W., Nishijo M., Ruiz P. (2013). Modeling cadmium exposures in low- and high-exposure areas in Thailand. Environ. Health Perspect..

[32] Yimthiang S., Pouyfung P., Khamphaya T., Kuraeiad S., Wongrith P., Vesey D.A., Gobe G.C., Satarug S. (2022). Effects of environmental exposure to cadmium and lead on the risks of diabetes and kidney dysfunction. Int. J. Environ. Res. Public Health.

[33] Bloch M.J., Basile J.N. (2013). Review of recent literature in hypertension: Updated clinical practice guidelines for chronic kidney disease now include albuminuria in the classification system. J. Clin. Hypertens (Greenwich).

[34] Hornung R.W., Reed L.D. (1990). Estimation of average concentration in the presence of nondetectable values. Appl. Occup. Environ. Hyg..

[35] Phelps K.R., Gosmanova E.O. (2020). A generic method for analysis of plasma concentrations. Clin. Nephrol..

[36] Zhu Y., Wang T., Jelsovsky J.Z. (2007). Bootstrap estimation of benchmark doses and confidence limits with clustered quantal data. Risk Anal..

[37] Slob W., Moerbeek M., Rauniomaa E., Piersma A.H. (2005). A statistical evaluation of toxicity study designs for the estimation of the benchmark dose in continuous endpoints. Toxicol. Sci..

[38] Slob W., Setzer R.W. (2014). Shape and steepness of toxicological dose-response relationships of continuous endpoints. Crit. Rev. Toxicol..

[39] Satarug S., Đorđević A.B., Yimthiang S., Vesey D.A., Gobe G.C. (2022). The NOAEL equivalent of environmental cadmium exposure associated with GFR reduction and chronic kidney disease. Toxics.

[40] Gburek J., Konopska B., Gołąb K. (2021). Renal handling of albumin-from early findings to current concepts. Int. J. Mol. Sci..

[41] Molitoris B.A., Sandoval R.M., Yadav S.P.S., Wagner M.C. (2022). Albumin uptake and processing by the proximal tubule: Physiological, pathological, and therapeutic implications. Physiol. Rev..

[42] Benzing T., Salant D. (2021). 2021. Insights into glomerular filtration and albuminuria. N. Engl. J. Med..

[43] Edwards A., Long K.R., Baty C.J., Shipman K.E., Weisz O.A. (2022). Modelling normal and nephrotic axial uptake of albumin and other filtered proteins along the proximal tubule. J. Physiol..

[44] Langelueddecke C., Roussa E., Fenton R.A., Wolff N.A., Lee W.K., Thévenod F. (2012). Lipocalin-2 (24p3/neutrophil gelatinase-associated lipocalin (NGAL) receptor is expressed in distal nephron and mediates protein endocytosis. J. Biol. Chem..

[45] Dizi E., Hasler U., Nlandu-Khodo S., Fila M., Roth I., Ernandez T., Doucet A., Martin P.Y., Feraille E., de Seigneux S. (2013). Albuminuria induces a proinflammatory and profibrotic response in cortical collecting ducts via the 24p3 receptor. Am. J. Physiol. Renal Physiol..

[46] Santoyo-Sánchez M.P., Pedraza-Chaverri J., Molina-Jijón E., Arreola-Mendoza L., Rodríguez-Muñoz R., Barbier O.C. (2013). Impaired endocytosis in proximal tubule from subchronic exposure to cadmium involves angiotensin II type 1 and cubilin receptors. BMC Nephrol..

[47] Gena P., Calamita G., Guggino W.B. (2010). Cadmium impairs albumin reabsorption by down-regulating megalin and ClC5 channels in renal proximal tubule cells. Environ. Health Perspect..

[48] Li L., Dong F., Xu D., Du L., Yan S., Hu H., Lobe C.G., Yi F., Kapron C.M., Liu J. (2016). Short-term, low-dose cadmium exposure induces hyperpermeability in human renal glomerular endothelial cells. J. Appl. Toxicol..

[49] Li Z., Jiang L., Tao T., Su W., Guo Y., Yu H., Qin J. (2017). Assessment of cadmium-induced nephrotoxicity using a kidney-on-a-chip device. Toxicol. Res..

