# Supplementary material for: Health Risk in a Geographic Area of Thailand with Endemic Cadmium Contamination: Focus on Albuminuria
Source: Toxics. 2023 Jan 11;11(1):68. doi: 10.3390/toxics11010068 (PMC9866753; doi:10.3390/toxics11010068)
Supplement: Supplementary file 1 [file toxics-11-00068-s001.zip › Correction SM_toxics-2136321.pdf]

toxics

MDPI

Supplemental Material: Health Risk in a Geographic Area of Thailand with Endemic Cadmium Contamination: Focus on Albuminuria

Soisungwan Satarug, David A. Vesey, Glenda C. Gobe, Supabhorn Yimthiang and Aleksandra Buha Đorđević

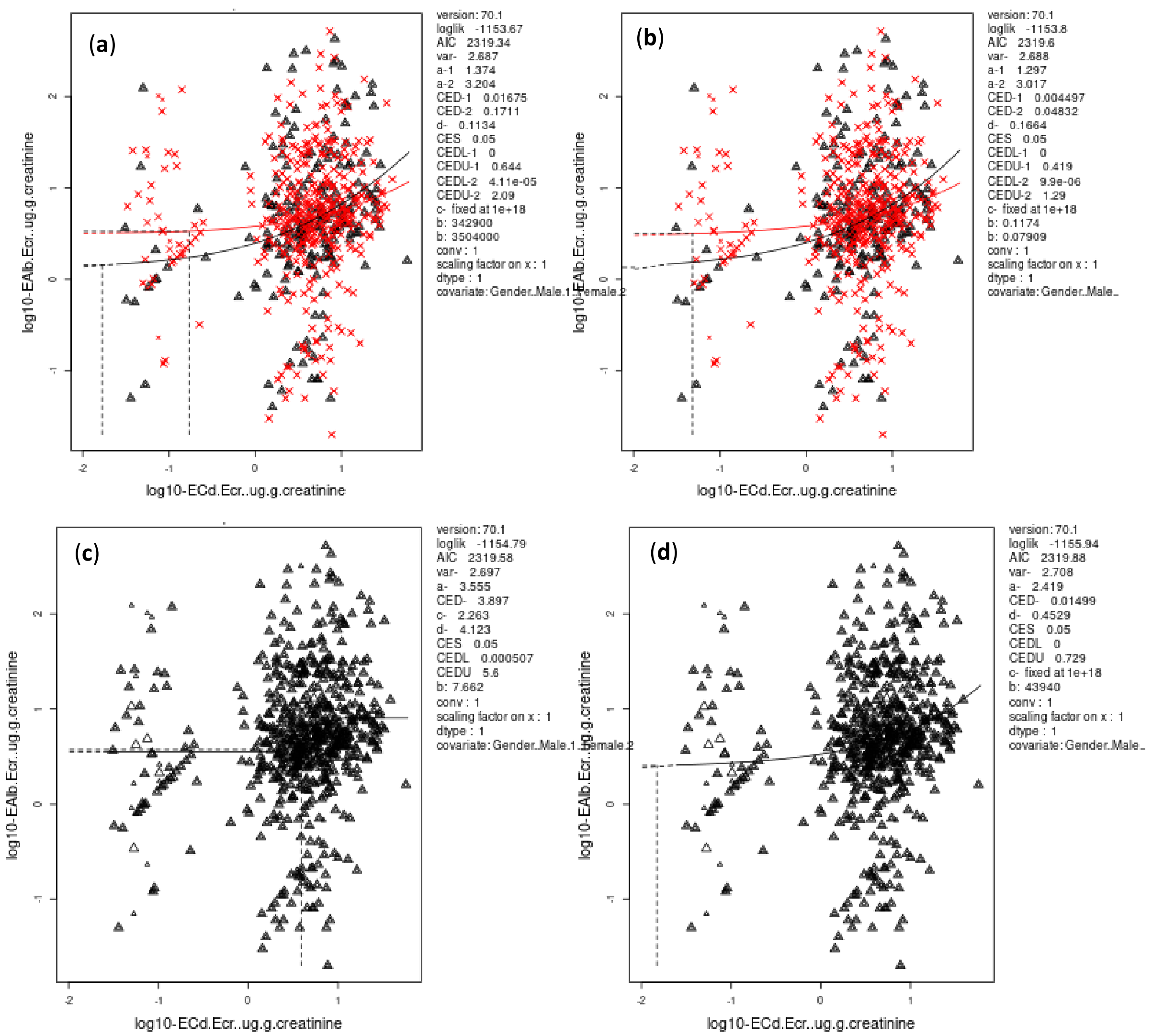

(e)

5% Increase of E_{alb}/E_{cr}	BMDL	BMDU	U/L
Men	4.16×10^{-6}	2.29	5.5×10^{5}
Women	3.89×10^{-4}	3.41	8.8×10^{3}

BMDL and BMDU values of E_{Cd}/E_{cr} were in µg/g creatinine. CI, confidence; U/L, BMDU/BMDL ratio.

Figure S1. Dose-effect relationship of E_{Cd}/E_{cr} and E_{alb}/E_{cr}. Data were fitted to 4 dose-response models; (**a**) an inverse exponential model; (**b**) a natural logarithmic model; (**c**) an exponential model; and (**d**) Hill model. The BMDL/BMDU values of E_{Cd}/E_{cr} corresponded to a 5% increase in E_{alb}/E_{cr} in men and women (**e**).

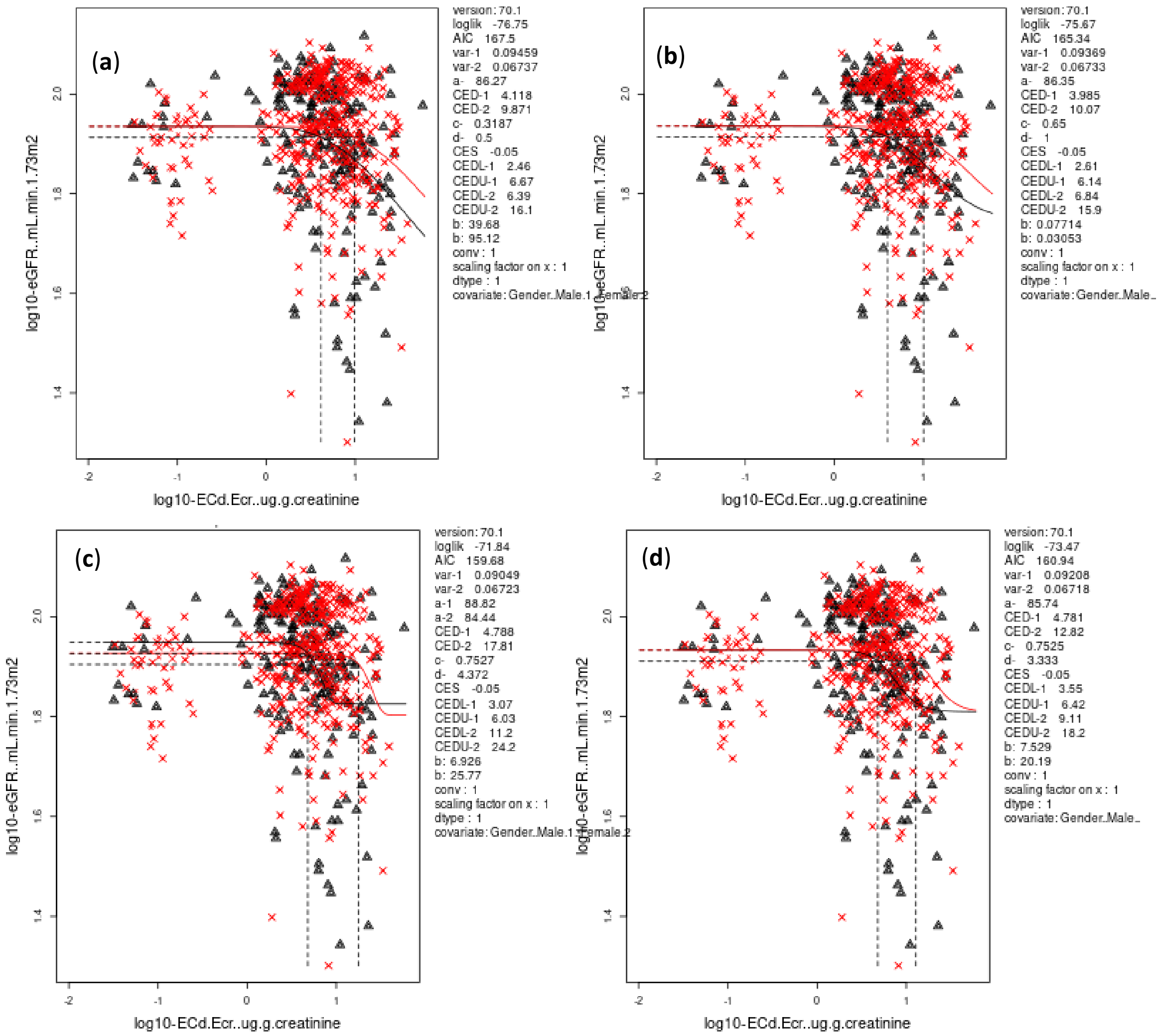

(e)

5% Decrease of eGFR	BMDL	BMDU	U/L
Men	2.07	6.93	3.35
Women	6.82	21.7	3.81

BMDL and BMDU values of E_{Cd}/E_{cr} were in µg/g creatinine.
CI, confidence interval; U/L, BMDU/BMDL ratio.

Figure S2. Dose-effect relationship of E_{Cd}/E_{cr} and eGFR. Data were fitted to 4 dose-response models; (**a)** an inverse exponential model; (**b**) a natural logarithmic model; (**c**) an exponential model; and (**d**) Hill model. The BMDL/BMDU values of E_{Cd}/E_{cr} corresponded to a 5% decrease of eGFR (**e**).

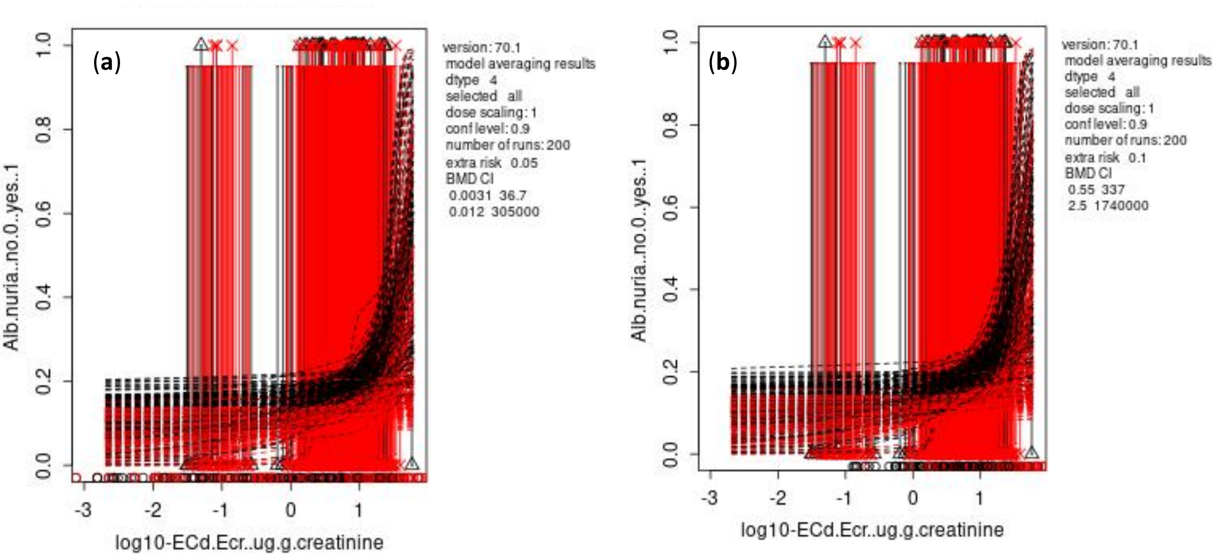

(**c**)

Endpoint	BMDL	BMDU	U/L
5% Prevalence of albuminuria			
Men	3.06×10^{-3}	36.7	1.2×10^{2}
Women	1.22×10^{-2}	3.05×10^{5}	2.5×10^{7}
10% Prevalence of albuminuria			
Men	0.55	337	612
Women	2.52	1.74×10^{6}	6.7×10^{5}

BMDL and BMDU values of E_{Cd}/E_{cr} were in µg/g creatinine. CI, confidence interval; U/L, BMDU/BMDL ratio. Albuminuria was defined as urinary albumin-to-creatinine ratios ≥ 20 mg /g for men, and ≥ 30 mg/g for women.

Figure S3. Dose-effect relationship of E_{Cd}/E_{cr} and albuminuria prevalence. Bootstrap model averaging of E_{Cd}/E_{cr} values corresponded to an increase of albuminuria prevalence by 5% (**a**) and 10% (**b**). BMDL/BMDU values of E_{Cd}/E_{cr} corresponded with 5% and 10% increases in prevalence of albuminuria (**c**).

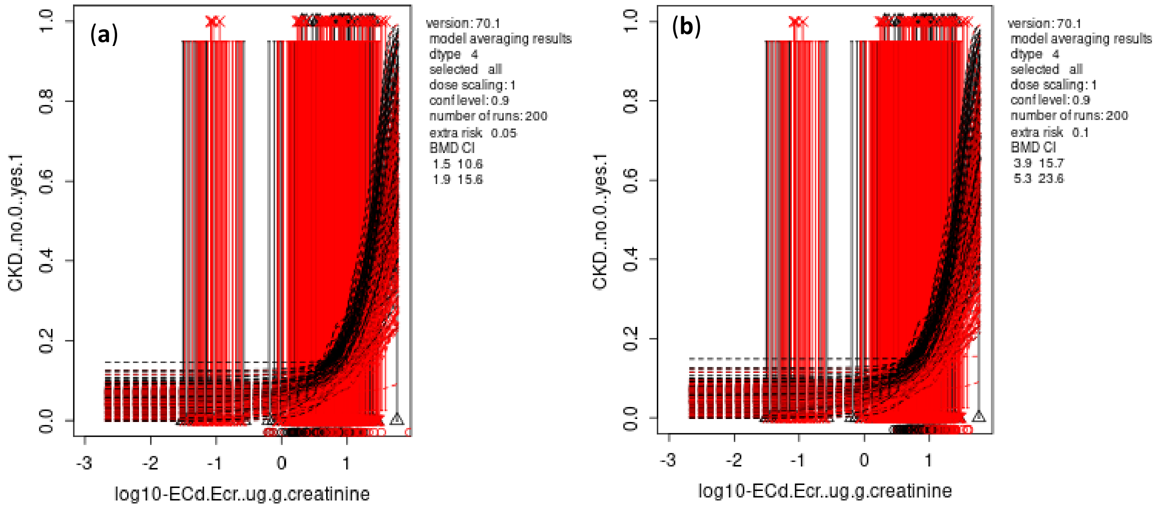

(c)

Endpoint	BMDL	BMDU	U/L
5% Prevalence of reduced eGFR			
Men	1.47	10.6	7.21
Women	1.93	15.6	8.08
10% Prevalence of reduced eGFR			
Men	3.92	15.7	4.00
Women	5.31	23.6	4.44

BMDL and BMDU values of E_{Cd}/E_{cr} were in µg/g creatinine. CI, confidence interval; U/L, BMDU/BMDL ratio. Reduced eGFR was defined as eGFR ≤ 60 mL/min/1.73 m^2.

Figure S4. Dose-effect relationship of E_{Cd}/E_{cr} and prevalence of reduced eGFR. Bootstrap model averaging of E_{Cd}/E_{cr} values corresponded to an increase of albuminuria prevalence by 5% (**a**) and 10% (**b**). The BMDL/BMDU values of E_{Cd}/E_{cr} corresponded with 5% and 10% increases in prevalence of albuminuria (**c**).

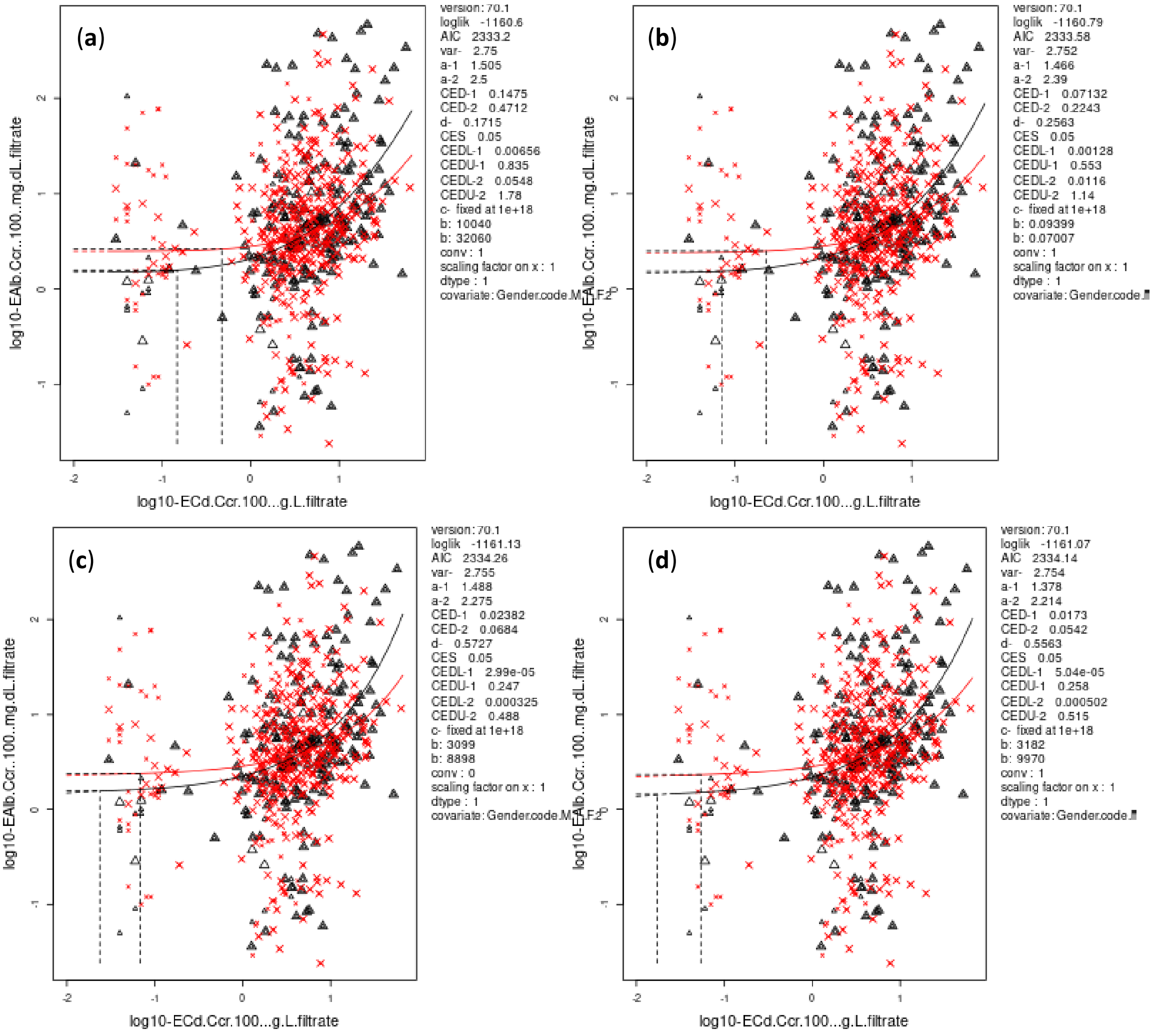

(e)

5% Increase of E_{alb}/C_{cr}	BMDL BMDU		U/L
Men	0.00053	1.11	2,094
Women	0.00357	2.09	585

BMDL and BMDU values of E_{Cd}/C_{cr} ×100 were in μg/L filtrate.

Figure S5. Dose-effect relationship of E_{Cd}/C_{cr} and E_{alb}/C_{cr}. Data were fitted to 4 dose-response models; (**a)** an inverse exponential model; (**b**) a natural logarithmic model; (**c**) an exponential model; and (**d**) Hill model. The BMDL/BMDU values of E_{Cd}/C_{cr} corresponded with a 5% increase in E_{alb}/C_{cr} (**e**).

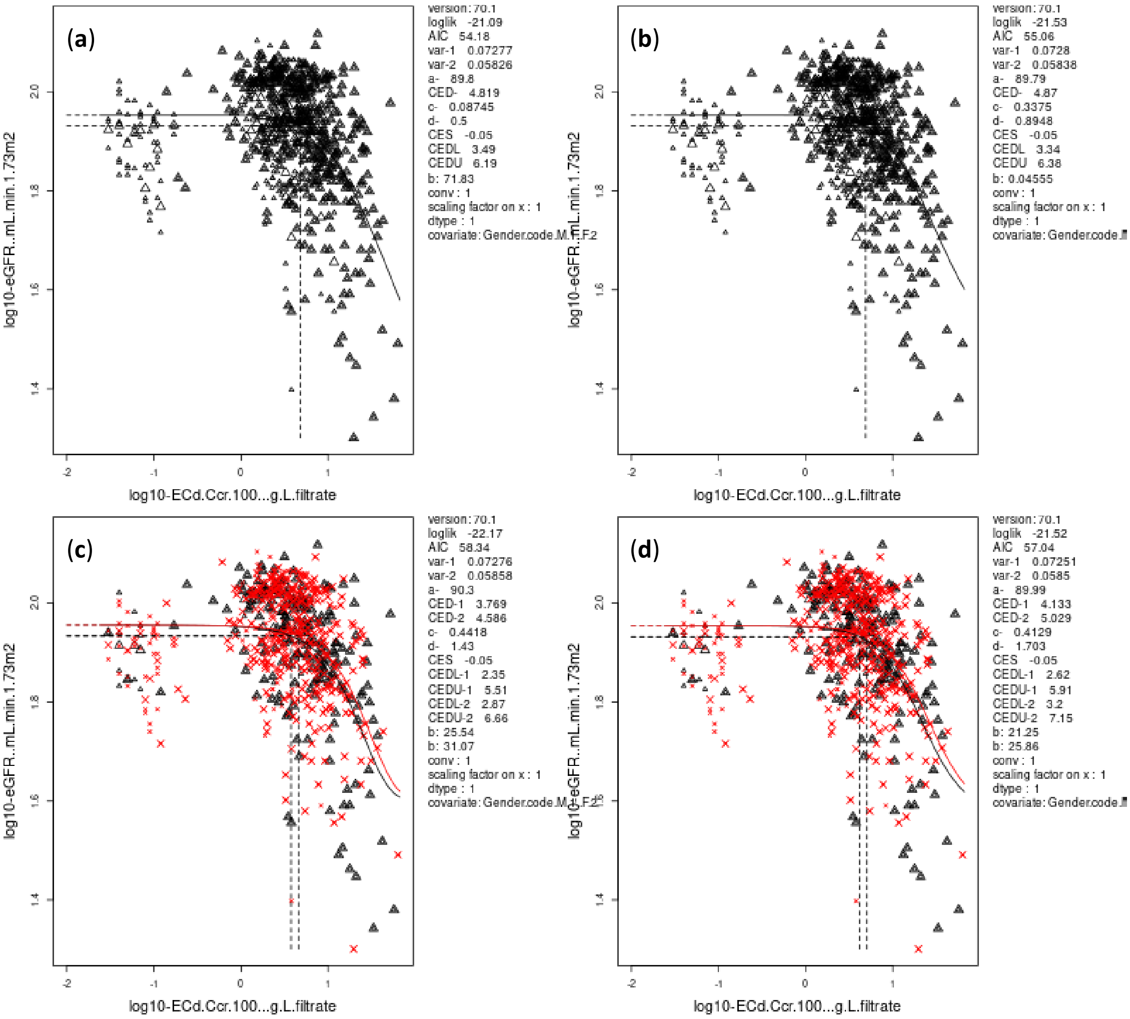

(e)

5% Decrease of eGFR	BMDL	BMDU	U/L
Men	2.15	6.71	3.12
Women	2.15	6.56	3.05

BMDL and BMDU values of E_{Cd}/C_{cr} ×100 were in µg/L filtrate.
CI, confidence interval; U/L, BMDU/BMDL ratio.

Figure S6. Dose-effect relationship of E_{Cd}/C_{cr} and eGFR. Data were fitted to 4 dose-response models; (**a**) an inverse exponential model; (**b**) a natural logarithmic model; (**c**) an exponential model; and (**d**) Hill model. The BMDL/BMDU values of E_{Cd}/C_{cr} corresponded to a 5% decrease of eGFR (**e**).

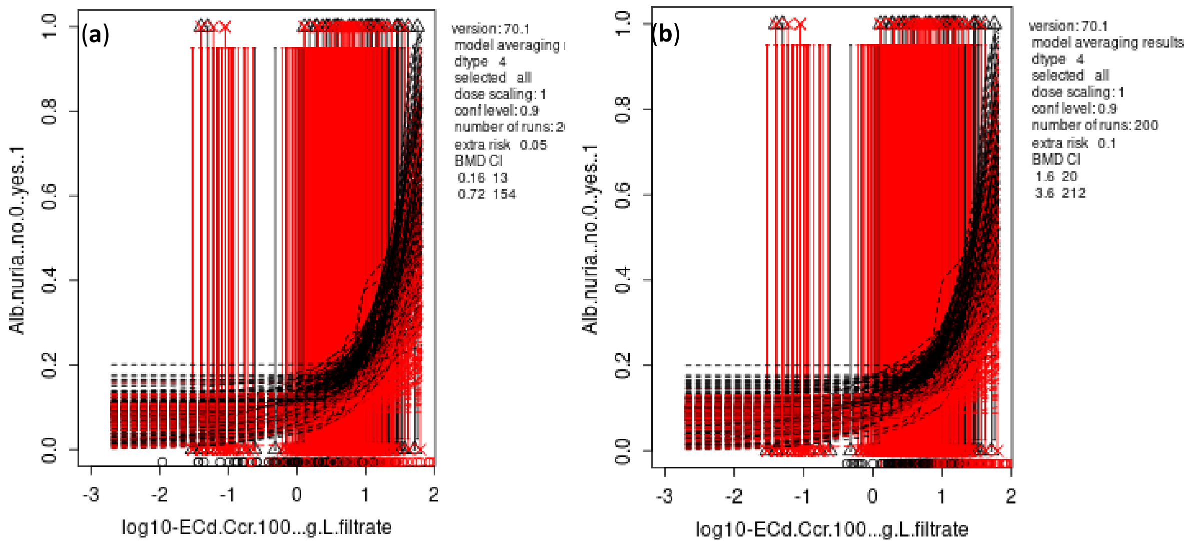

(**c**)

Endpoint	BMDL	BMDU	U/L
5% Prevalence of albuminuria			
Men	0.163	13	80
Women	0.718	154	214
10% Prevalence of albuminuria			
Men	1.65	20	12
Women	3.55	212	60

BMDL and BMDU values of E_{Cd}/C_{cr} were in µg/L filtrate. CI, confidence interval; U/L, BMDU/BMDL ratio. Albuminuria was defined as $E_{alb}/C_{cr} \times 100 \geq 20$ mg/L filtrate for men, and ≥ 30 mg/L for women.

Figure S7. Dose-effect relationship of E_{Cd}/C_{cr} and albuminuria prevalence. Bootstrap curves for BMD model averaging of E_{Cd}/C_{cr} values corresponded to an increase of albuminuria prevalence by 5% (**a**) and 10% (**b**). BMDL/BMDU values of E_{Cd}/C_{cr} corresponded with 5% and 10% increases in prevalence of albuminuria (**e**).

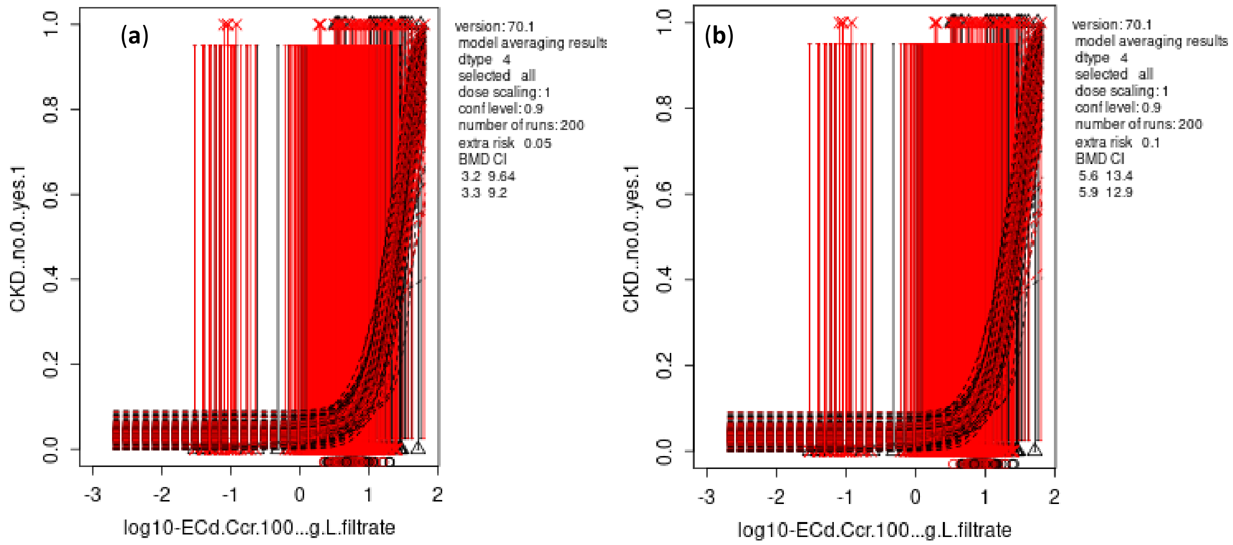

(**c**)

Endpoint	BMDL	BMDU	U/L
5% Prevalence of reduced eGFR			
Men	3.22	9.64	2.99
Women	3.33	9.2	2.76
10% Prevalence of reduced eGFR			
Men	5.61	13.4	2.39
Women	5.88	12.9	2.19

BMDL and BMDU values of E_{Cd}/C_{cr}×100 were in µg/L filtrate. CI, confidence interval; U/L, BMDU/BMDL ratio. Reduced eGFR was defined as eGFR ≤ 60 mL/min/1.73 m^2.

Figure S8. Dose-effect relationship of E_{Cd}/C_{cr} and prevalence of reduced eGFR. Bootstrap curves for model averaging of E_{Cd}/C_{cr} values corresponded to an increase of prevalence of reduced eGFR by 5% (**a**) and 10% (**b**). The BMDL/BMDU values of E_{Cd}/C_{cr} corresponded with 5% and 10% increases in prevalence of reduced eGFR (**c**).
